# Acute Paraplegia due to Thoracic Hematomyelia

**DOI:** 10.1155/2016/3138917

**Published:** 2016-07-12

**Authors:** Aykut Akpınar, Bahattin Celik, Ihsan Canbek, Ergun Karavelioğlu

**Affiliations:** ^1^Department of Neurosurgery, Haseki Research and Training Hospital, Aksaray Street, Fatih, 34130 Istanbul, Turkey; ^2^Department of Neurosurgery, Özel Osm Ortadoğu Hospital, 63100 Şanlıurfa, Turkey; ^3^Department of Neurosurgery, Afyon Kocatepe University Hospital, 03103 Afyonkarahisar, Turkey

## Abstract

Spontaneous intraspinal intramedullary hemorrhage is a rare entity with the acute onset of neurologic symptoms. The etiology of idiopathic spontaneous hematomyelia (ISH) is unknown, and there are few published case reports. Hematomyelia is mostly associated with trauma, but the other nontraumatic etiologies are vascular malformations, tumors, bleeding disorders, syphilis, syrinx, and myelitis. MRI is a good choice for early diagnosis. Hematomyelia usually causes acute spinal cord syndrome due to the compression and destruction of the spinal cord. A high-dose steroid treatment and surgical decompression and evacuation of hematoma are the urgent solution methods. We present idiopathic spontaneous hematomyelia of a previously healthy 80-year-old male with a sudden onset of back pain and paraplegia.

## 1. Introduction

The ISH is a rare entity. Trauma is considered the most common cause of hematomyelia. The other etiologies are vascular malformations, cavernomas, aneurysms, primary tumors or metastasis, syrinx, meningomyelitis, myelitis and arachnoiditis, hemophilia, Von Willebrand's disease, factor 11 deficiency, anticoagulant therapy, and other rare causes including syphilis and aortic aneurysms [1–4]. Surgical intervention is to be performed as early as possible for pain relief and preservation or restoration of the neurological function [1–9].

## 2. Case Report

An 80-year-old man developed sudden severe back pain with no history of trauma, and then he tried to walk. However, due to weakness and numbness in his legs, he could not walk and collapsed to the ground. He presented to an outside hospital with paraplegia, and then he was transferred to a tertiary center for further management. On admission, neurological examination of his motor power was reduced (0/5) in both lower extremities (paraplegia), tendon reflexes in both lower limbs had increased, Babinski responses were positive, there was anesthesia/hypoesthesia for light touch, and there was hypoesthesia below the thoracal (T5-T6) dermatome. His knee- and ankle-jerk reflexes were absent bilaterally. There were urine and stool incontinence. The complete blood count (hemoglobin, hematocrit, and platelet count), biochemistry profile (kidney function, liver function, proteins, and glucose), prothrombin time, and INR were normal, and he did not have additional comorbidities (hypertension, diabetes mellitus, etc.). In addition, he did not use an anticoagulant therapy. At the time of admission to emergency, only subcutaneous low molecular weight heparin (0.3 mL) was conducted. A high-dose steroid treatment was given on the day of his admission (bolus 30 mg/kg administered over 15 minutes with maintenance infusion of 5.4 mg/kg per hour infused for 23 hours).

**Figure 1:**
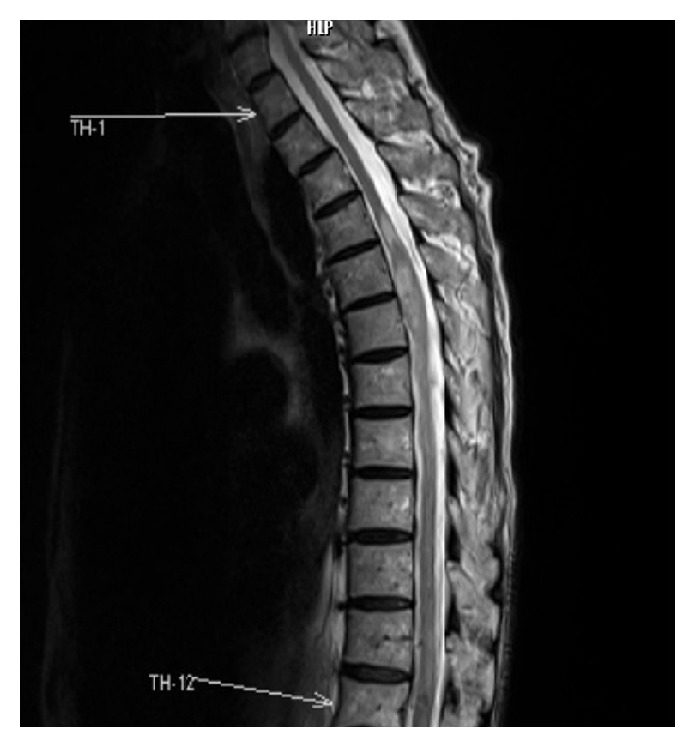
Magnetic resonance imaging (MRI) revealed an intramedullary mass with signal intensity surrounded by a zone of high and low signal on T2 weighted images.

**Figure 2:**
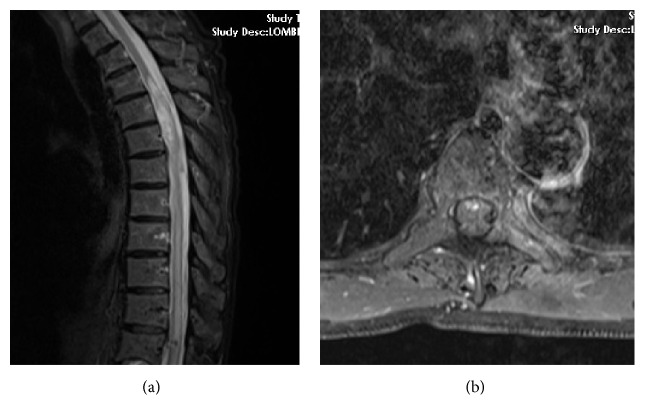
Magnetic resonance imaging scans revealed intramedullary hematoma and syrinx cavity.

The Computed Tomography (CT) scan was not diagnostically helpful. Magnetic resonance imaging (MRI) revealed an intramedullary mass with signal intensity surrounded by a zone of high and low signals on both T2 weighted images (Figures 1, 2(a), and 2(b)). Unfortunately, because of the lack of time, we did not have contrast-enhanced MRI or spinal angiography, so we could not eliminate the vascular pathologies. Because of mass effect, there was obstruction of cerebrospinal fluid and syrinx. After written informed consent was obtained from the patient and his family, an operation was performed.

**Figure 3:**
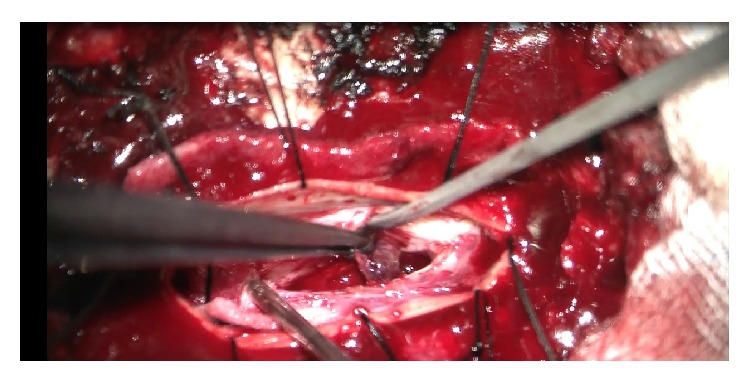
Intraoperative findings showing the hematoma after cordotomy.

The patient underwent the operation on the day of admission. After thoracic vertebra 5 and 6 laminectomy, microscopic dural opening was revealed, a small paramedian myelotomy was performed at the level, and blunt dissection and aspiration of the hematoma were done. A hematoma was completely removed (Figure 3). There were no vascular abnormalities and tumors. The pathology report confirmed the diagnosis of intramedullary hemorrhage. After the operation, there was no recovery of sensory or motor functions, and he began receiving rehabilitation treatment.

## 3. Discussion

Hematoma in the spinal cord is called hematomyelia. Spontaneous hematomyelia is a rare entity. The etiologies are trauma, vascular malformations (AVM, cavernomas, and aneurysms), primary tumors or metastasis, and parenchymal abnormalities, such as syrinx, meningomyelitis, myelitis and arachnoiditis, or spinal radiation changes, and bleeding disorders (hemophilia, Von Willebrand's disease, factor 11 deficiency, and anticoagulant therapy). Other rare causes include syphilis, aortic aneurysm, hemorrhagic myelitis or hyperdynamic states of spinal artery circulation, and cardiovascular disorders (hypertension, heart failure, and atherosclerosis). Trauma is considered the most common cause of hematomyelia [3, 6–9].

The idiopathic hematomyelia's real etiology is unknown [1–5, 9]. In this case, the patient had no coagulopathy, tumor, or vascular pathology.

MRI examination is a better diagnostic tool than conventional myelography or CT. The final diagnosis of ISH is often confirmed by microscopy [4, 6].

Early diagnosis and surgery are essential for cases of ISH in the literature review. Previously reported cases of idiopathic spontaneous intraspinal intramedullary hematoma presented with acute, subacute, or chronic myelopathy. Acute hematomyelia is often accompanied by sudden severe back or neck pain and then presents with myelopathy syndromes due to the mass effect's acute obstruction of CSF and blood in the spine. Hematomyelia usually presents with an acute onset and rapid deterioration in neurologic status [6]. Prognostic factors after spinal cord compression is diagnosed are strongly related to the time interval between symptom onset and surgical decompression [1, 5].

There are currently no guidelines for the management of acute hematomyelia, and treatment is typically directed toward the underlying cause [7].

The conservative management is unknown in idiopathic hematomyelia; therefore, surgical removal of hematoma is the treatment of choice for acutely deteriorated idiopathic hematomyelia [10].

Early recognition, accurate diagnosis, and appropriate treatment can decrease morbidity and improve the prognosis and outcome for patients with IMH [2, 5].

Even though high-dose steroid treatment, early decompression, and hematoma microdissection were performed, our patient's neurological examination was unchanged. We should conduct the operation as early as possible, but, first, physical and neurological examination must be done at an emergency department. We must eliminate the blood tests for cardiac or pulmonary emergencies, and then CT and MRI should be done, and when we discover the pathology, we should request the patient's or his family's permission; all these levels took time. We were in the theater room in the 16th hour of the patient's first admission.

Given the limited number of similar reports, whether the stabbing pain preceded the intramedullary hemorrhage remains to be determined [1–10].

The present case report clearly describes the spontaneous occurrence of hematomyelia without demonstrable predisposing or precipitating cause, which is not clearly established in the literature. The patient suffered acute and severe neurological deterioration and has limited postoperative functional recovery as a result of the hemorrhage. Given the devastating clinical course of our patient, it is important to recognize the possibility of spontaneous spinal intramedullary hemorrhage.

## 4. Conclusion

Nontraumatic idiopathic spontaneous intraspinal intramedullary hemorrhage is a very rare entity. Intramedullary hemorrhages are catastrophic. Early recognition, diagnosis, medical treatment, and urgent surgery can decrease morbidity. Using high-dose steroid treatment and microscopic hematoma evacuation may decrease morbidity.
